# Editorial: Post-translational Modification in Response to Stresses in Bacteria

**DOI:** 10.3389/fmicb.2022.844854

**Published:** 2022-02-22

**Authors:** Vijay Pancholi

**Affiliations:** Department of Pathology, The Ohio State University, Columbus, OH, United States

**Keywords:** PTM, metabolic stresses, post-translational modifications, bacteria, reversible phosphorylation, phosphoproteomics, Ser/Thr/Tyr kinases, Ser/Thr/Tyr phosphatases

## Role of PTMs in Bacteria in Response to Stress: a Multifunctional and Dynamic Modulatory Mechanism Beyond Genetic Confinement

Post-translational modification (PTM) of proteins is a genetic confinement-free essential biochemical change that occurs during protein translation when a cell is ready to execute its function. PTMs also occur in response to various extra and intracellular environmental cues for the cell survival and fitness required for cell growth and development. Bacteria face a variety of nutritional and environmental stresses which can impact their survival while in contact with inert abiotic (inert surface) or in association with the living host cell. PTMs of proteins, therefore, are dynamic, reversible, or irreversible, which may occur sequentially or at the same time at one or many vulnerable side chains (hydroxyl, amino, or thiol) of amino acids (serine, threonine, tyrosine, histidine, aspartate, asparagine, lysine, arginine, and cysteine), bringing structural and functional changes into the affected proteins fitting to the spatial physiological status of the cell (Macek et al., 2019). Such combinatorial effects impact bacterial morphology, metabolism and fitness, drug susceptibility pattern, biofilm formation, latency, spore formation, and virulence. Intra- and inter-protein cross-talks between two different PTMs lead to additional complex fine-tuning steps to perform complex biological functions. PTM machinery, as recently classified, consists of enzymatic and substrate-specific “Writers” (kinases, ubiquitin ligases, acetyltransferase, and glycosylases that modify the proteins by transferring specific chemical groups), “Erasers” (Phosphatases, deubiquitinases, deacetylase, deglycosylase that confer reversibility by catalyzing the removal of modified groups), and a complex enzymatic or non-enzymatic “Readers,” which are dependent on a specific pre-existing PTM showing combinatorial dependency (Positive or negative Ubiquitination that is dependent on phosphorylation, sumoylation, ADP-ribosylation, or methylation) (Leutert et al., 2021). With the advent of qualitative and quantitative mass-spectrometry analysis and increasingly sophisticated instrumentation with integrated robust bioinformatics software and genomic and proteomic databases, accurate identification of these PTM(s) occurring at a single amino acid resolution is now possible to obtain (Choudhary and Mann, 2010; Macek et al., 2019; Leutert et al., 2021). The precise interpretations and physiological relevance of many of these PTMs occurring in a signaling network forms transiently or permanently in a small population of a given molecule still depend on the dynamic range and the efficiency of the given mass-spectrometer (Choudhary and Mann, 2010; Olsen and Mann, 2013; Macek et al., 2019; Leutert et al., 2021).

Among several PTMs in bacteria described so far (Macek et al., 2019), phosphorylation-mediated PTM is unquestionably the most extensively studied and is primarily mediated by two-component-system (TCS), phosphoenolpyruvate: carbohydrate transport system (PTS), bacterial protein-tyrosine (BY-kinase), and Ser/Thr kinases (Hank's/eukaryote-type) with mono or dual specificity. In addition, a new class of kinases, UbK (Ubiquitous kinase), also has been described (Nguyen et al., 2017). The presence of four recent investigations on the “Post-translational modification in response to stresses in bacteria” includes four different bacteria, each focusing on different PTMs with hitherto undescribed novel functions yet providing a shared and convergent theme.

The study by Sultan et al. in this series supports the recent findings that the bacterial metabolic enzymes are the significant phosphorylation sites by cellular Ser/Thr/Tyr kinases (Schastnaya et al., 2021). By employing phosphoproteomic and biochemical approaches, this study has identified impact on the phosphoproteome of *Escherichia coli* in the absence of hitherto uncharacterized *E. coli* ser/thr kinase (YeaG). The mutant lacking YeaG displayed a distinct phenotype of significantly shorter lag phase and a metabolic shift from glucose to malate resulting in differential phosphorylation in central carbon metabolism and transporters. Various biochemical and genomic approaches in progress may explain the observed differential modulation mechanism. In another report, Pang et al. provide the first global acetylome features of a Gram-negative food-borne pathogen, *Vibrio vulnificus*. The global proteomic analysis of this strain revealed acetylation in 40% of the total proteins, significantly enriched in factors involved in multiple central metabolic processes, virulence, and drug resistance, thus providing an insightful role of this PTM in *V. vulnificus* virulence and drug resistance. Similarly to this PTM, Zhao et al. has provided comprehensive succinylome profiling in *Staphylococcus epidermidis*, a commensal Gram-positive bacterium known for its role in biofilm formation and causing drug-resistant nosocomial infection by escaping from the immune elimination and drug resistance. The authors provide evidence as to why succinylation-mediated PTM plays a regulatory role in various physiological processes. Their global proteomic analysis-based succinylome study reveals enrichment of this PTM once again in energy metabolism and quorum sensing essential pathophysiological processes, including biofilm formation. While these omic-based studies have identified crucial players modulated by specific PTMs, their physiological relevance awaits *in vivo* studies and focused protein chemistry and enzymological analysis. Kant and Pancholi in their publication in this series, revisit the prevailing assumption that tyrosine phosphorylation, unlike Ser/Thr phosphorylation (Jin and Pancholi, 2006; Agarwal et al., 2011; Kant et al., 2015), does not exist in *Streptococcus pyogenes*. This Gram-positive human pathogen causes various extracellular invasive and often fatal skin and respiratory diseases, many of which may result in debilitating post-streptococcal autoimmune sequelae. This study provides the first detailed characterization of tyrosine kinase identified previously in *S. pneumoniae* as UbK (Ubiquitous kinase) (Pelletier et al., 2019). *In vitro* and *in vivo* phosphoproteomic analysis has also identified important targets involving *S. pyogenes'* central metabolism and virulence regulation. RNA-seq-based global transcriptome analysis of the mutant lacking this kinase reveals altered expression levels of genes belonging to the central carbohydrate metabolism, PTS, and transport metabolism. These changes are reflected in *S. pyogenes* ability to adhere to and invade host cells, modulate host inflammatory responses, and form biofilms, which together determine the disease outcomes.

Together, all these publications reveal the following salient findings. (i) Proteomic and genomic approaches play a critical role in understanding the potential and impactful modulatory role of PTMs in bacterial phenotypes, metabolism, virulence, and antibiotic susceptibility. (ii) All investigations reveal that the stress-induced PTMs primarily target central metabolism, the impact of which in turn result subsequently in the form of modulated phenotypic and functional changes, including altered cell division, virulence, drug resistance, and/or biofilm formation. (iii) The observed finely tuned cellular functions seem to involve dynamic cross-talks among various kinases targeting the same substrate. Hence, *in vivo* biochemical validation of PTMs is essential to understand the physiological relevance of the *in vitro*-observed PTMs.

## Author Contributions

VP conceived the concept and reviewed all manuscripts, analyzed the result, and concluded the theme.

## Funding

The present study was supported in part by OSU Department of Pathology internal funding grant #502171.

## Conflict of Interest

The author declares that the research was conducted in the absence of any commercial or financial relationships that could be construed as a potential conflict of interest.

## Publisher's Note

All claims expressed in this article are solely those of the authors and do not necessarily represent those of their affiliated organizations, or those of the publisher, the editors and the reviewers. Any product that may be evaluated in this article, or claim that may be made by its manufacturer, is not guaranteed or endorsed by the publisher.
